# Molecular Characterization of Grass Carp GIPR and Effect of Nutrition States, Insulin, and Glucagon on Its Expression

**DOI:** 10.1155/2022/4330251

**Published:** 2022-11-07

**Authors:** Guokun Yang, Xiaomin Liang, Yanle Jiang, Chengquan Li, Yanmin Zhang, Xindang Zhang, Xulu Chang, Yawei Shen, Xiaolin Meng

**Affiliations:** ^1^College of Fisheries, Henan Normal University, Xinxiang 453007, China; ^2^Engineering Technology Research Center of Henan Province for Aquatic Animal Cultivation, Henan Normal University, Xinxiang 453007, China

## Abstract

GIP plays an important regulatory role in glucose and lipid metabolism. As the specific receptor, GIPR is involved in this physiological process. To assess the roles of GIPR in teleost, the GIPR gene was cloned from grass carp. The ORF of cloned GIPR gene was 1560 bp, encoding 519 amino acids. The grass carp GIPR was the G-protein-coupled receptor which contains seven predicted transmembrane domains. In addition, two predicted glycosylation sites were contained in the grass carp GIPR. The grass carp GIPR expression is in multiple tissues and is highly expressed in the kidney, brain regions, and visceral fat tissue. In the OGTT experiment, the GIPR expression is markedly decreased in the kidney, visceral fat, and brain by treatment with glucose for 1 and 3 h. In the fast and refeeding experiment, the GIPR expression in the kidney and visceral fat tissue was significantly induced in the fast groups. In addition, the GIPR expression levels were markedly decreased in the refeeding groups. In the present study, the visceral fat accumulation of grass carp was induced by overfed. The GIPR expression was significantly decreased in the brain, kidney, and visceral fat tissue of overfed grass carp. In primary hepatocytes, the GIPR expression was promoted by treatment with oleic acid and insulin. The GIPR mRNA levels were significantly reduced by treatment with glucose and glucagon in the grass carp primary hepatocytes. To our knowledge, this is the first time the biological role of GIPR is unveiled in teleost.

## 1. Introduction

Glucose-dependent insulinotropic polypeptide (GIP) is an incretin hormone which is released into the circulation following nutrient ingestion [1]. The crucial role of GIP is stimulating insulin release from pancreatic islet *β* cells [2, 3]. Moreover, GIP also increases lipogenesis in adipose tissue, promotes bone formation, and induces proliferation of hippocampal progenitor cells [2]. GIP exerts its roles by binding to its specific receptor, namely, GIPR [4]. The GIPR was firstly cloned from the cerebral cortex cDNA library of rat in 1993 [2, 5, 6] and was followed cloned in the hamster [5, 7] and human [5, 8]. The GIPR is a glycoprotein, which belongs to the secretin/vasoactive intestinal peptide (VIP) family of receptors. In this protein family, it includes receptors for glucagon-likepeptide-1 (GLP-1), VIP, secretin, pituitary adenylate cyclase activating polypeptide (PACAP), and glucagon [6].

GIPR is a seven transmembrane protein, which is a member of G-protein-coupled receptor (GPCR) superfamily [2, 5, 9]. As the GPCR, GIPR has a large N-terminal extracellular domain which is vital to receptor activation and high-affinity GIP binding [6, 10]. The C-terminal cytoplasmic domain of GIPR is associated with intracellular signaling [2, 10]. Moreover, the first transmembrane domain of GIPR is essential for cAMP coupling [10]. In addition, the conserved N-glycosylation sequence (N-X-S/T) is located in the N-terminal of GIPR [6]. And the C-terminal and the third cytoplasmic loop of GIPR contain many potential phosphorylation sites [6]. GIPR mRNA expression is a wide range of tissues in the human and mouse [2, 6]. The report reveals that the GIPR is detected in the adipose tissue, kidney, heart, bone, intestine, pancreas, and several regions of the central nervous system (CNS) [2, 6, 11]. In human islets, GIPR expression is detected in *α*, *β*, *δ*, and *γ* cells [11, 12]. Furthermore, GIPR expression is extensive in the rodent brain, such as the cerebral cortex, hippocampus, brain stem, cerebellum, and olfactory bulb of rats [5, 11]. In mice, GIPR expression level is reduced with an age-increased dependent [13].

The zebrafish GIP (zfGIP) can activate the zebrafish glucagon receptor [14] and human GLP-1 receptor [15]. However, as an endogenous receptor of GIP, GIPR is essential for GIP playing its biological functions. For example, the GIPR signaling deficiency or gain regulates food intake in mice, which mediates by the control of leptin sensitivity [16–18]. Moreover, the diet-induced obesity is alleviated via reducing adipose tissue mass in the mice of GIPR knockout or antagonism [19, 20]. The PI3K/Akt and PKA signaling pathways are involved in the biological roles of GIP binding GIPR. In pancreatic *β* cells, GIP increases insulin secretion by binding GIPR, in which the intracellular cyclic AMP (cAMP) level promotes and activates PKA signaling pathway [21, 22]. Furthermore, GIP promotes *β* cell survival by inhibiting apoptotic protein Bax expression which mediates the PI3K/Akt signaling pathway [21, 23]. In adipose tissue, GIP increases glucose transporter 4 (GLUT4) and lipoprotein lipase (LPL) expression and promotes hormone-sensitive lipase (HSL) activity by activating the PKA signaling pathway [21, 24]. In addition, GIPR mediates protein kinase G (PKG) signaling pathway to promote activation and phosphorylation of HSL [21, 25].

As the incretin, GIP plays an important role in lipogenesis, insulin secretion, and bone formation [2], which GIPR is involved in the regulatory functions [21]. Our previous study indicates that GIP takes part in glucose and lipid metabolism of grass carp [22]. However, the roles of fish GIPR have been rarely reported. To investigate the functions of GIPR in fish, the grass carp GIPR was isolated from brain tissue. The tissue-specific expression of GIPR was evaluated by real-time PCR. The effects of OGTT and fast and refeeding on GIPR expression were tested. The visceral fat accumulation of grass carp was induced by overfeeding. The GIPR expression was assessed in the overfed grass carp. In vitro, the effects of glucose, oleic acid, insulin, and glucagon on GIPR mRNA levels were assessed. To our knowledge, this study is the first report of GIPR function of fish.

## 2. Materials and Methods

### 2.1. Animals

In this study, the grass carp was obtained from Yanjin Fishery (Yanjin County, Henan Province). Before the experiment, fish were domesticated for two weeks at indoor tanks. The water quality parameters for fish acclimation were controlled as follows: temperature, 26–28°C; dissolved oxygen concentration, 5.5–6.2 mg/L; and pH 7.2–7.5. The fish were fed commercial pellets (Tongwei, China) of three times per day (8:30, 13:30, and 18:30). All animal experiments were approved by the Animal Care Committee of Henan Normal University.

### 2.2. Molecular Identification and Sequence Analysis of Grass Carp GIPR

The RT-PCR (reverse transcription PCR) was performed to clone grass carp GIPR in this study. Before the experiment, the zebrafish GIPR (XM 005157739.4) sequence was used to blast the predicted sequence of GIPR in the NCBI Transcriptome Shotgun Assembly Sequence database of grass carp (https://www.ncbi.nlm.nih.gov). The specific primers for GIPR cloning were shown in Table 1. Then, the total RNA of grass carp brain was obtained by RNAiso Plus (Takara, Japan). The PrimeScript RT reagent kit was used to synthesize the first-strand cDNA. The PCR program used for GIPR cloning was as follows: 94°C for 3 min, 35 cycles of 94°C for 30 s, 56°C for 30 s, 72°C for 2 min, 72°C for 5 min, and 4°C for infinity. After purifying with E.Z.N.A Gel Extraction Kit (OMEGA, Biotek), the PCR fragments were ligated to the pMD19-T vector (Takara, Japan). The cloned GIPR was analyzed based on sequencing result. The SignalP server-5.0 (http://www.cbs.dtu.dk/services/SignalP/) was used to predict the signal peptide of grass carp GIPR. The glycosylation sites of grass carp GIPR were analyzed by NetNGlyc 1.0 Server (http://www.cbs.dtu.dk/services/NetNGlyc/). The transmembrane domains of grass carp GIPR were analyzed by TMHMM server 2.0 (http://www.cbs.dtu.dk/services/TMHMM/). The protein motif of grass carp GIPR was predicted by the Simple Modular Architecture Research Tool (http://smart.emblheidelberg.de/). The spatial structure of grass carp GIPR was analyzed by Swiss-model (https://swissmodel.expasy.org/). Sequence alignments were performed by ClustalW2 software (http://www.ebi.ac.uk/Tools/msa/clustalo/). The phylogenetic tree of GIPR was constructed with MEGAX by the neighbor-joining method.

### 2.3. Tissue-Specific Expression and Effects of OGTT and Fast and Refeeding on GIPR Expression

In the tissue expression experiment, three grass carp were acclimated for two weeks. Then, fish were anesthetized and sacrificed by decapitation. The experimental samples (the telencephalon, mesencephalon, cerebellum, hypothalamus, pituitary, head kidney, kidney, heart, liver, spleen, foregut, midgut, hindgut, fat, muscle, gonad, and gill) were collected and snap-frozen in liquid nitrogen. The collected samples were stored at −80°C until RNA extraction.

In the OGTT, the experimental process was referred to the previous study [26, 27]. The grass carp were domesticated for two weeks. In the glucose treatment group, fish were performed by gavage glucose solution with the concentration of 1.67 mg/g BW (body weight). In the control group, the fish were performed with PBS. After treatment by gavage for 1, 3, and 6 h, the fish were anesthetized and sacrificed by decapitation. The brain, kidney, and visceral fat were collected and snap-frozen in liquid nitrogen (*n* = 8/group). The collected samples were stored at −80°C until RNA extraction.

In the fast and refeeding experiment, the experimental procedure was referred to the previous study [27, 28]. After acclimating for two weeks, the experiment was implemented. In the control group (feeding), fish were fed for 14 days. In the fasting group (fast), the fish were fast for 14 days. In the refeeding group (refeeding), the fish were fast for 14 days and were refed before 6 h for sampling on day 14. By the end of the study, the fish were anesthetized and sacrificed by decapitation. The kidney and visceral fat were quickly collected and snap-frozen in liquid nitrogen (*n* = 12/group). The collected samples were stored at −80°C until RNA extraction.

### 2.4. Overfed-Induced Visceral Fat Accumulation of Grass Carp and GIPR Expression

To evaluate the effect of fat accumulation on GIPR mRNA levels in grass carp, the grass carp was induced by overfed. Grass carp were purchased from a fish farm (Yanjin, Henan). Fish were acclimated in a recirculating aquaculture system and fed basic diets for two weeks. Healthy fish were distributed into 6 tanks (150 L) with 20 fish per tank (three tanks per treatment). During the 6-week experimental period, fish were fed commercial feed thrice daily at 08:30, 13:30, and 18:30. In the control group (control), fish were fed at a rate of about 3% body weight every day. In the overfed-induced group (induced), fish were fed until not eating every time. The body weight of fish was recorded every two weeks, and the amount of feed was adjusted based on the body weight.

After 6-week feeding trial, four fish from each tank were chosen to be sampled. Four fish from each tank were chosen to be measured body weight and the weights of visceral adipose tissues to calculate the visceral adipose ratio (VAR) (VAR, % = (final body weights (g)/visceral adipose weight (g) × 100). Blood samples were collected from the caudal vein of each fish. The blood sample was incubated at 4°C at least for 1 h. After centrifugation of 10 min at 7500 g, serum was collected and stored at -80°C for measure contents of glucose and TG. The contents of serum glucose and TG were determined with commercial kits (Jiancheng, China). Then, the kidney, brain, and visceral fat samples were collected from four fish in each tank and quickly frozen in liquid nitrogen for RNA isolation.

### 2.5. Grass Carp Primary Hepatocyte Isolation and Treatments

The experimental method of primary hepatocyte isolation was referred to a previous study [27, 29]. The isolated hepatocytes were cultured in the 24-well plate with 1 mL DMEM/F12 medium contained 10% fetal bovine serum (FBS) with the density of 8 × 10^5^ cells/well. After overnight culture, the cell medium was replaced to fresh DMEM/F12 without FBS. Before treatment, the hepatocytes were cultured for 1 h in the DMEM/F12 without FBS. (1) The hepatocytes were treated with glucose (35 mM) or oleic acid (80 *μ*g/mL) for 12 and 24 h. (2) The hepatocytes were treated with human insulin or glucagon at the dose of 0, 10, 100, and 1000 nM for 3 and 6 h. By the end of the study, the hepatocytes were lysed by RNAiso Plus for RNA extraction.

### 2.6. RNA Extraction, Reverse Transcription, and Real-Time PCR

The total RNA of all samples was extracted by the RNAiso Plus. The concentrations of total RNA were detected by the UV spectrophotometer (Nanodrop 2000, Thermo, USA). The gDNA Eraser was used to digest the genomic DNA from 1 *μ*g of total RNA at 42°C for 2 min. Then, the PrimeScript RT reagent kit (PrimeScript RT reagent kit with gDNA Eraser, Takara, China) was used to synthesize the 1st-strand cDNA. In the real-time PCR, the 1st-strand cDNA was used as the template. The used primers were listed in Table 1. Real-time PCR was performed on a LightCycler 480II Sequence Detection System (Roche, Rotkreuz, Switzerland) using the SYBR Green PCR Master Mix (Bimake, Shanghai, China). The procedure of real-time PCR was as follows: 95°C for 5 min, 40 cycles of 95°C for 15 s, 56°C for 15 s, and 72°C for 30 s. 18S rRNA or *β*-actin was used as the internal reference. The results of gene mRNA levels were normalized to that of internal reference genes using the comparative Ct method [30].

### 2.7. Statistical Analyses

All data of this study are represented as mean ± S.E.M. The SPSS version 18.0 (SPSS Inc., Chicago, IL, USA) was used to perform statistical analysis. The data were analyzed with the unpaired Student *t*-test (two-group comparisons) or one-way ANOVA (multigroup comparisons) to determine the statistical significance of differences between the groups. It was considered significant that the probability value was of *P* < 0.05.

## 3. Results

### 3.1. Molecular Characterization of Grass Carp GIPR

**Figure 1:**
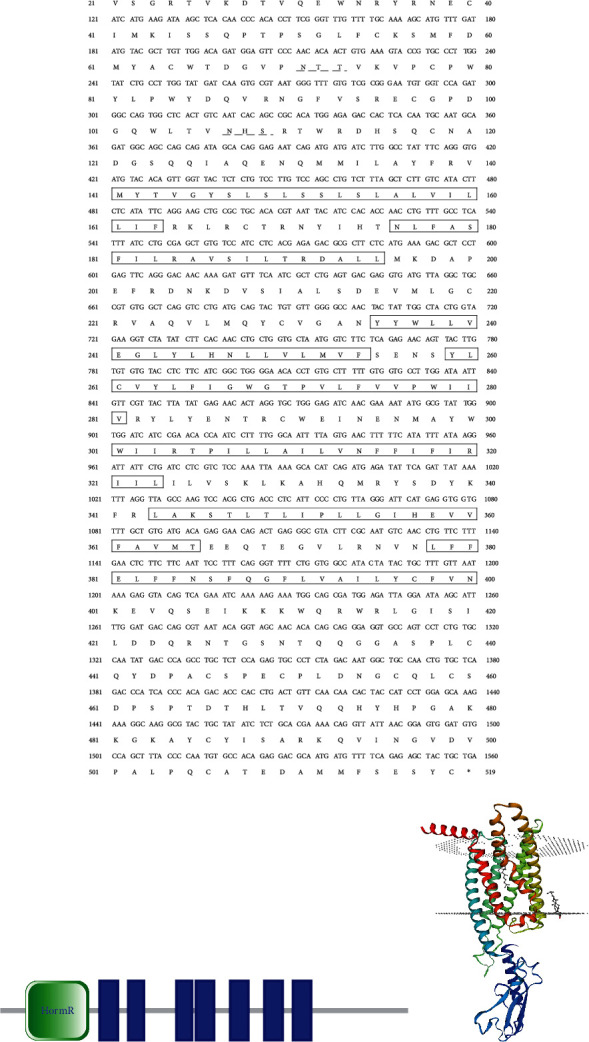
(a) The cDNA and deduced amino acids of grass carp GIPR. Single underlined represent signal peptides; box represent transmembrane domains; dotted lines represent N-linked glycosylation sites; the asterisk represents the stop codon. (b) The domain organization of grass carp GIPR was predicted by SMART. (c) The predicted three-dimensional structure of grass carp GIPR. The model 7fin.1.A was used as the reference model for the predicted spatial structure.

**Figure 2:**
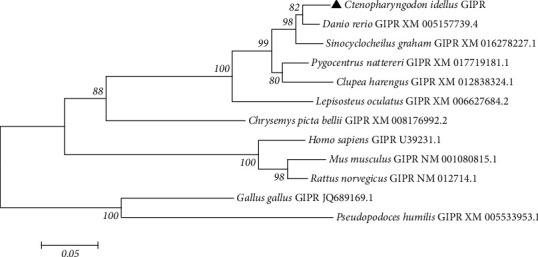
Phylogenetic tree based on amino acid alignment for GIPR in different species.

The ORF of cloned GIPR was 1560 bp, encoding 519 amino acids (Figure 1(a)). The first 19 amino acid was the predicted signal peptide. The analysis result by TMHMM server 2.0 revealed that the GIPR was the classical GPCR, which had seven transmembrane domains with the intracellular N-terminal and extracellular C-terminal (Figure 1(a)). Moreover, the predicted results of protein motif and spatial structure were indicated that the grass carp GIPR was the seven transmembrane protein (Figures 1(b) and 1(c)). In grass carp GIPR, two predicted N-glycosylation sites were located in the intracellular N-terminal (Figure 1(a)). The result of sequence alignment showed that grass carp GIPR displayed high identities to that of *Danio rerio* (92.02%), *Sinocyclocheilus grahami* (90.56%), and *Pygocentrus nattereri* (82.27%) (Table 2). The phylogenetic tree was constructed with GIPR sequences of various species. The results revealed that the various fishes were clustered into one clade with high bootstrap values (Figure 2).

### 3.2. Tissue-Specific Expression of Grass Carp GIPR

**Figure 3:**
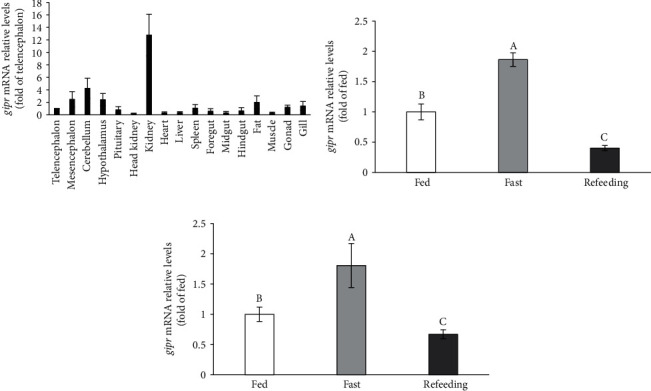
Analysis the expression pattern of GIPR in grass carp. (a) Tissue distribution of GIPR in grass carp. The mRNA levels were quantified by real-time PCR. All data were represented as the mean ± S.E.M. (*n* = 3). (b, c) Effects of fast and refeeding on the GIPR expression in grass carp. Effects of fast and refeeding on GIPR expression. The mRNA expression of GIPR in the kidney (b) and visceral fat (c) of grass carp was quantified by real-time PCR. The results were represented as the fold of fed. All data are shown as mean ± S.E.M. (*n* = 10–12). Significant differences (*P* < 0.05) were indicated by different letters.

The tissue distribution of GIPR was evaluated by the real-time PCR. The results revealed that the mRNA transcripts of GIPR were detected in all detected tissues of grass carp. The most abundant expression level of GIPR was detected in the kidney, brain regions, and visceral fat tissue of grass carp (Figure 3(a)).

### 3.3. Effects of OGTT and Fast and Refeeding on GIPR Expression

**Figure 4:**
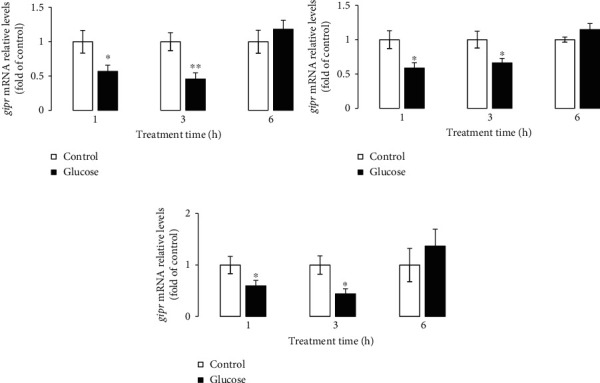
Effects of OGTT on the GIPR expression in grass carp. The mRNA expression of GIPR in the kidney (a), visceral fat (b), and brain (c) of grass carp was quantified by real-time PCR. The results were represented as the fold of control. All data are shown as mean ± S.E.M. (*n* = 7–8). ^∗^*P* < 0.05, ^∗∗^*P* < 0.01.

To assess the effects of energy state on the mRNA transcripts of grass carp GIPR, the OGTT and fast and refeeding experiments were performed. In the fast and refeeding experiments, the GIPR mRNA levels were dramatically promoted in the kidney and visceral fat tissue of the fast group. Moreover, the GIPR expression was markedly reduced in the kidney and visceral fat tissue of refeeding group than that in the fed and fast groups (Figures 3(b) and 3(c)). In the OGTT experiment, the GIPR mRNA levels were observably inhibited in the kidney, visceral fat, and brain by treatment with glucose for 1 and 3 h (Figure 4).

### 3.4. Overfed-Induced Visceral Fat Accumulation of Grass Carp and GIPR Expression

**Figure 5:**
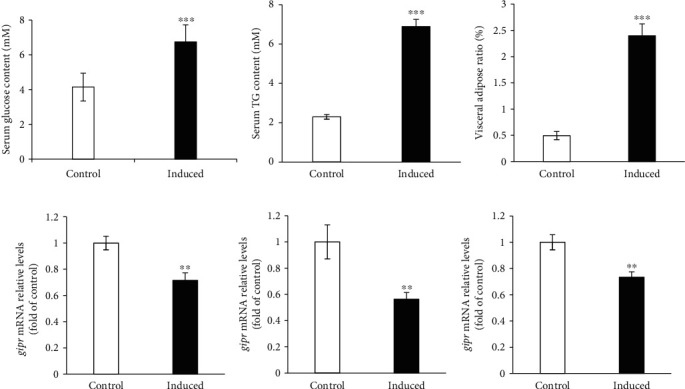
Overfed-induced visceral fat accumulation of grass carp and GIPR expression. (a, b) The serum glucose (a) and TG (b) contents of control and overfed-induced groups. (c) The VAR of control and overfed-induced groups. (d–f) The mRNA expression of GIPR in the kidney (d), visceral fat (e), and brain (f) of grass carp was quantified by real-time PCR. The results were represented as the fold of control. All data are shown as mean ± S.E.M. (*n* = 10–12). ^∗∗^*P* < 0.01, ^∗∗∗^*P* < 0.001.

As shown in Figure 5, the serum glucose and TG contents were observably promoted in the overfed-induced group (Figures 5(a) and 5(b)). The VAR was also significantly promoted in the induced group (Figure 5(c)). Moreover, the fat was observably accumulated in the abdominal cavity of the induced group (Supplemental Figures 1A, 1B). The GIPR expression in the visceral fat, kidney, and brain tissues was observably reduced in the induced group (Figures 5(d)–5(f)).

### 3.5. Effects of Glucose, Oleic Acid, Insulin, and Glucagon on GIPR Expression in Hepatocytes

**Figure 6:**
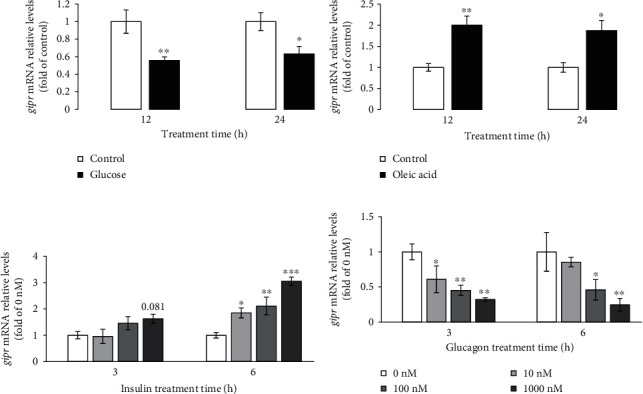
Effects of glucose, oleic acid, insulin, and glucagon on GIPR expression in grass carp primary hepatocytes. (a, b) Effects of glucose and oleic acid on GIPR expression in grass carp primary hepatocytes. (c, d) Effects of insulin and glucagon on GIPR expression in grass carp primary hepatocytes. The cells were seeded in 24-well plates at 8 × 10^5^ per well in 1 mL DMEM/F12 with 10% FBS. The next day, cells were placed in DMEM/F12 without FBS for 1 h serum starvation. (1) For the glucose and oleic acid studies, the cells were treated with glucose (35 mM) or oleic acid (80 *μ*g/mL) for 12 and 24 h. (2) For the insulin and glucagon studies, the cells were treated with human insulin or human glucagon at doses of 0, 10, 100, and 1000 nM for 3 and 6 h. All data are shown as mean ± S.E.M. (*n* = 5 − 6). ^∗^*P* < 0.05, ^∗∗^*P* < 0.01, and ^∗∗∗^*P* < 0.001.

In primary hepatocytes, the GIPR expression levels were memorably reduced by treatment with glucose for 12 and 24 h. Moreover, the GIPR mRNA levels were dramatically induced by treatment with oleic acid for 12 and 24 h (Figures 6(a) and 6(b)). By treatment with insulin, the GIPR expression was markedly induced in primary hepatocytes for 6 h. However, the GIPR expression levels were significantly decreased in primary hepatocytes by treatment with glucagon for 3 and 6 h (Figures 6(c) and 6(d)).

## 4. Discussion

As the incretin, GIP is involved in many important physiological functions, in which the GIPR plays important roles [2, 10]. To assess the roles of GIPR in fish, the GIPR was cloned from grass carp brain in our study. The grass carp GIPR is a classical GPCR and is the seven transmembrane proteins with the intracellular N-terminal and extracellular C-terminal. The protein structure of grass carp GIPR is similar to that of mammalian GIRP which is seven transmembrane protein belonging to the VIP/secretin family of receptors [6]. The pervious study indicated that the GIPR had a large N-terminal extracellular domain containing a consensus N-glycosylation sites [6, 10]. The N-terminal domain of the GIPR is vital for high-affinity GIP binding [2, 6, 10]. Moreover, the N-terminal domain of GIPR is necessary for receptor activation and cAMP coupling [2, 6, 10]. In the grass carp GIPR, the intracellular N-terminal is relatively large and also contains two predicted N-glycosylation sites. It speculates that the intracellular N-terminal of grass carp GIPR may take part in GIP binding and receptor activation. The alignment result showed that grass carp GIPR displayed high identities to that of *Danio rerio* (92.02%), *Sinocyclocheilus grahami* (90.56%), and *Pygocentrus nattereri* (82.27%). Furthermore, the phylogenetic tree result revealed that the various fishes were clustered into one clade with high bootstrap values. Based on those results, the cloned sequence in our study is the grass carp GIPR sequence.

The GIPR was firstly identified in transplantable insulinoma and insulin-secreting *β* cell line of hamster [10]. Subsequently, the rat GIPR was cloned from cerebral cortex cDNA library [5, 10]. In the present study, the grass carp GIPR expression is in multiple tissues. The high transcriptional level of grass carp GIPR was detected in the kidney, brain regions, and fat tissue. The result is similar to previous studies. In mammals, the GIPR expression was also detected in the multiple tissues, including intestine, adipose tissue, pituitary, heart, spleen, kidney, and several regions in the CNS [2, 6, 10]. The results indicate that the GIPR wide expression in multiple tissues is a universal phenomenon.

In the OGTT experiment, the GIPR mRNA level was memorably decreased by treatment with glucose for 1 and 3 h. Furthermore, the results of our previous studies showed that the grass carp serum glucose levels were significantly promoted by treatment with glucose for 1 and 3 h in the OGTT experiment [26, 29, 31]. The previous studies showed that the Zucker diabetic fatty (ZDF) rats were with extreme hyperglycemia, and the mRNA and protein levels of GIP receptor were significantly downregulated [32, 33]. Moreover, the reduced GIPR expression levels in ZDF rats were relieved following normalization of hyperglycemia by phlorizin treatment [32]. In woman, the GIPR expression in the subcutaneous fat was negatively correlated with fasting blood glucose [34]. The researcher suggested that the hyperglycemia-induced downregulation of GIPR expression may be closely associated with ubiquitination [10, 35]. In the present study, the GIPR mRNA level was also memorably inhibited with glucose treatment in grass carp primary hepatocytes. Similarly, GIPR level in INS (832/13) cells was strongly decreased by glucose treatment with the time- and concentration-dependent manner [36]. In addition, the protein levels of GIPR were reduced in rat and human islets exposed to glucose [35]. These results reveal that glucose level is the vital regulatory factors of GIPR mRNA and protein expression.

Fasting and refeeding are used to investigate the biological response of teleosts [37]. In the present study, the GIPR mRNA level in the kidney and fat tissue of grass carp was markedly induced by fasting for 14 days. Moreover, the GIPR mRNA level was reduced in the refeeding group. The roles of GIP receptor were closely related to the nutritional status. In the ZDF rats, the levels of GIP receptor mRNA and protein were decreased than that of lean rats [10, 32, 33]. In obese nondiabetic women, the GIPR level was dramatically decreased in adipose tissue [34]. However, the GIPR expression was induced in ECs which were stressed by the removal of serum from the culture media [38]. Furthermore, high-fat diet induces to increased adipocyte mass in normal mice, whereas fed high-fat diets in GIPR^(−/−)^ mice will not induce obese [10]. In addition, the fatty acid (palmitate) markedly promoted the GIPR transcriptional level in the islets isolated from lean Zucker rats, INS (832/13) cell line, and BRIN-D11 *β* cells [36]. Similarly, the GIPR transcriptional level in grass carp hepatocyte was also induced by treatment with fatty acid in our study. The above results indicate that nutritional status plays important role in regulation of GIPR expression.

In our study, the serum glucose, TG, and VAR were significantly promoted in the overfed-induced group. Similarly, the previous studies indicated that the serum glucose and TG levels were also observably promoted in the overfeeding-induced groups [39–42]. Moreover, the numbers of lipid droplets in liver tissue were also increased in the overfeeding zebrafish [39, 41–43]. In addition, the overfeeding-induced zebrafish had more adipocytes accumulated in the abdominal cavity [44, 45]. Furthermore, visceral adipocytes were markedly larger in the obese group [42]. And our present study also showed that the visceral adipocytes were observably larger in the overfed-induced group. Based on those results, the visceral fat accumulation of grass carp was successfully induced with overfed in our study. The GIPR expression was significantly inhibited in the induced group in the present study. The previous studies showed that the GIPR mRNA and protein were observably decreased in the obese rats and women [32–34]. And the GIPR expression was markedly reduced in the hyperglycemic rats [36]. The decreased GIPR expression in the overfed-induced grass carp may be the response to the high serum glucose level in our study.

As the important endocrine cytokine, insulin and glucagon are involved in many physiological processes. In our study, the GIPR transcriptional level in grass carp primary hepatocyte was observably inhibited by treatment with insulin and was significantly induced by treatment with glucagon. A previous study revealed that GIPR^(-/-)^ mice had impaired glucose tolerance and significantly reduced insulin gene expression and secretion compared with wild-type mice [10, 46, 47]. Similarly, the GIPR expression level in visceral fat of postmenopausal nondiabetic women was positively correlated with fasting insulin [34]. However, the culture medium addition of insulin can inhibit GIPR expression in the arterial smooth muscle cells [38]. These results reveal that the insulin is closely related to the GIPR expression level. It is rarely reported that the interactive correlation is between GIPR and glucagon. Glucagon is hyperglycemic *in vivo* in many fish species and induces glucose production in isolated hepatocytes [48]. The reason of glucagon reduced GIPR expression in our study may be the promoted glucose levels in grass carp hepatocytes by glucagon induction. And the regulation mechanism needs to be elucidated in future study.

In conclusion, the grass carp GIPR was cloned in our study. The GIPR transcriptional level was detected in all detected tissues and with high levels in the kidney, brain regions, and fat tissue of grass carp. A study of OGTT experiment showed that GIPR transcriptional level was dramatically inhibited by glucose treatment. In the fast and refeeding experiment, the GIPR mRNA levels were dramatically induced in the fast groups and were markedly reduced in the refeeding groups. In the overfed-induced grass carp, the GIPR transcriptional level was markedly reduced. In the grass carp hepatocyte, the GIPR transcriptional level was reduced by treatment with glucose and glucagon and was increased by treatment with oleic acid and insulin. To our knowledge, this study is the first biological report of GIPR in teleost.

## Figures and Tables

**Table 1:** Primers used in this study.

Name	Sequences (5′—3′)	Purpose	Amplicon size (bp)	Accession no.
ORF-F	ATGAAGAGCACCTCTGCCAT	ORF clone	1560	
ORF-R	TCAGCAGTAGCTCTCTGAA			
GIPR-qRT-F	TGCTGGTGCTAATGGTC	GIPR real-time PCR	180	
GIPR-qRT-R	GGATTGGTGTTCGGATG			
*18S*-F	ATTTCCGACACGGAGAGG	Reference gene	90	EU047719
*18S*-R	CATGGGTTTAGGATACGCTC			
*β-Actin*-F	TATGTTGGTGACGAGGCTCA	Reference gene	127	M25013
*β-Actin*-R	GCAGCTCGTTGTAGAAGGTG			

**Table 2:** Identities of grass carp GIPR compared with other species.

Species	Identity
*Homo sapiens*	U 39231.1 55.32%
*Mus musculus*	NM 001080815.1 54.82%
*Rattus norvegicus*	NM 012714.1 55.98%
*Gallus gallus*	JQ689169.1 60.75%
*Pseudopodoces humilis*	XM 005533953.1 49.29%
*Chrysemys picta bellii*	XM 008176992.2 57.29%
*Danio rerio*	XM 005157739.4 92.02%
*Pygocentrus nattereri*	XM 017719181.1 82.27%
*Sinocyclocheilus grahami*	XM 016278227.1 90.56%
*Lepisosteus oculatus*	XM 006627684.2 71.71%
*Clupea harengus*	XM 012838324.1 77.28%
*Ctenopharyngodon idellus*	100%

## Data Availability

The data that support the findings of this study are available from the corresponding author upon reasonable request.

## Abbreviations

GIP: Glucose-dependent insulinotropic polypeptide
GIPR: GIP receptor
ORF: Open reading frame
OGTT: Oral glucose tolerance test
VIP: Vasoactive intestinal peptide
GLP-1: Glucagon-like peptide-1
PACAP: Pituitary adenylate cyclase activating polypeptide
GPCR: G-protein-coupled receptor
CNS: Central nervous system
zfGIP: Zebrafish GIP
cAMP: Cyclic AMP
GLUT4: Glucose transporter 4
LPL: Lipoprotein lipase
HSL: Hormone-sensitive lipase
PKG: Protein kinase G
RT-PCR: Reverse transcription PCR
B.W: Body weight
FBS: Fetal bovine serum
ZDF: Zucker diabetic fatty
VAR: Visceral adipose ratio.

## Supplementary Materials

Supplementary Materials Supplemental figure 1: the fat tissue morphology in abdominal cavity of control (A) and overfed-induced (B) groups.
