# Supplementary material for: Molecular Characterization of Grass Carp GIPR and Effect of Nutrition States, Insulin, and Glucagon on Its Expression
Source: Aquac Nutr. 2022 Nov 7;2022:4330251. doi: 10.1155/2022/4330251 (PMC9973162; doi:10.1155/2022/4330251)
Supplement: Supplementary Materials — Supplemental figure 1: the fat tissue morphology in abdominal cavity of control (A) and overfed-induced (B) groups. [file 4330251.f1.pdf]

A

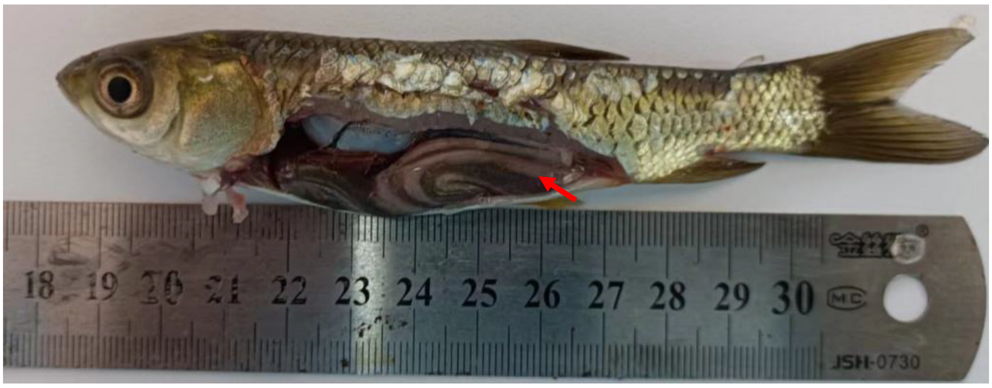

B

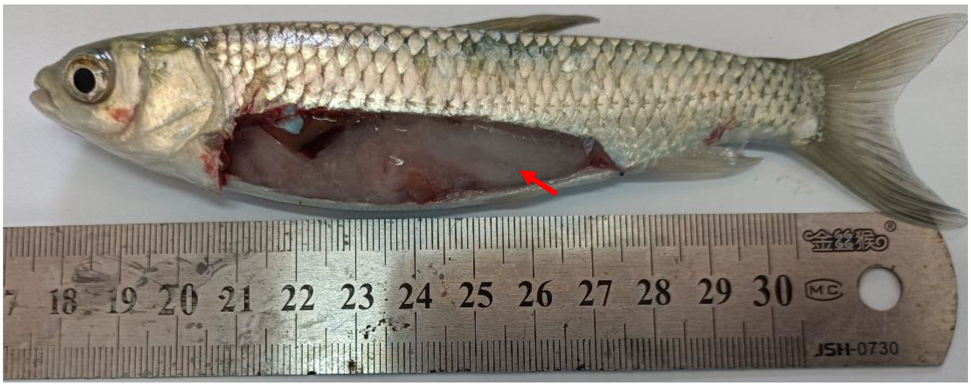

Supplemental figure 1. The fat tissue morphology in abdominal cavity of control (A) and overfed induced (B) groups.
